# Association of the habitual dietary intake with the fatty liver index and effect modification by metabotypes in the population-based KORA-Fit study

**DOI:** 10.1186/s12944-024-02094-0

**Published:** 2024-04-04

**Authors:** M. Schepp, D. Freuer, N. Wawro, A. Peters, M. Heier, D. Teupser, C. Meisinger, J. Linseisen

**Affiliations:** 1 University of Augsburg, University Hospital Augsburg, EpidemiologyAugsburg, Germany; 2 Institute of Epidemiology, Helmholtz Zentrum München – German Research Center for Environmental Health (GmbH), Neuherberg, Germany; 3 Chair of Epidemiology, Institute for Medical Information Processing, Biometry and Epidemiology, Medical Faculty, Ludwig-Maximilians-Universität München, Munich, Germany; 4 German Center for Diabetes Research (DZD), Munich-Neuherberg, Germany; 5 KORA Study Centre, University Hospital Augsburg, Augsburg, Germany; 6 Institute of Laboratory Medicine, University Hospital, LMU Munich, Munich, Germany; 7 Institute for Medical Information Processing, Biometry and Epidemiology, Medical Faculty, Ludwig-Maximilians-Universität München, Munich, Germany

**Keywords:** Diet, Fatty liver index, Food groups, Carbohydrates, Metabotype

## Abstract

**Background:**

Non-alcoholic fatty liver disease (NAFLD) is an emerging threat for public health with diet being a major risk factor in disease development and progression. However, the effects of habitual food consumption on fatty liver are still inconclusive as well as the proposed role of the individuals’ metabolic profiles. Therefore, the aim of our study is to examine the associations between diet and NAFLD with an emphasis on the influence of specific metabotypes in the general population.

**Methods:**

A total of 689 participants (304 men and 385 women) of the KORA-Fit (S4) survey, a follow-up study of the population-based KORA cohort study running in the Region of Augsburg, Germany, were included in this analysis. Dietary information was derived from repeated 24-h food lists and a food frequency questionnaire. The intake of energy and energy-providing nutrients were calculated using the national food composition database. The presence of fatty liver was quantified by the fatty liver index (FLI), and metabotypes were calculated using K-means clustering. Multivariable linear regression models were used for the analysis of habitual food groups and FLI; for the evaluation of macronutrients, energy substitution models were applied.

**Results:**

A higher consumption of nuts and whole grains, and a better diet quality (according to Alternate Healthy Eating Index and Mediterranean Diet Score) were associated with lower FLI values, while the intake of soft drinks, meat, fish and eggs were associated with a higher FLI. The isocaloric substitution of carbohydrates with polyunsaturated fatty acids was associated with a decreased FLI, while substitution with monounsaturated fatty acids and protein showed increased FLI. Statistically significant interactions with the metabotype were observed for most food groups.

**Conclusion:**

The consumption of plant-based food groups, including nuts and whole grains, and diet quality, were associated with lower FLI values, whereas the intake of soft drinks and products of animal origin (meat, fish, eggs) were associated with a higher FLI. The observed statistically significant interactions with the metabotype for most food groups could help to develop targeted prevention strategies on a population-based level if confirmed in independent prospective studies.

**Supplementary Information:**

The online version contains supplementary material available at 10.1186/s12944-024-02094-0.

## Background

Non-alcoholic fatty liver disease (NAFLD) is a metabolic disorder which is characterized by an excess accumulation of fat in liver cells, and often times develops on the basis of existing obesity; this hepatic steatosis is strongly linked with insulin resistance and disturbances in glucose and lipid metabolism; thus, it is nowadays called metabolic dysfunction-associated fatty liver disease (MAFLD). If untreated, MAFLD may develop to non-alcoholic steatohepatitis (NASH) and promote the development of metabolic and cardiovascular diseases, eventually leading to increased early mortality [1–3]. During the past three decades it is estimated that the global MAFLD prevalence increased exponentially, and currently affects about one in three people; thus MAFLD became a major global health concern [3]. Despite its substantial public health impact, MAFLD is rarely addressed in national health agendas of most countries though there is a pressing need for effective prevention strategies [4]. Lifestyle modifications leading to weight loss are currently recommended to counteract the progression of MAFLD but there is still inconclusive data regarding the recommendation of a specific dietary pattern [5, 6]. Food-based dietary guidelines are commonly used in public health communication because of their simplicity and practicability for the general population compared to nutrient-based recommendations [7]. However, while the intake of specific nutrients such as fructose have shown to detrimentally affect MAFLD [8], the influence of specific food items has not been extensively researched yet, especially on a population-based level [9]. Therefore, our study aims to examine associations between the habitual consumption of different food groups in the general population and fatty liver disease as defined by the fatty liver index (FLI); this index was established and validated in 2006 by Bedogni et al. [10]. Additionally, we applied metabotyping, which is a procedure that stratifies subjects into most homogenous subgroups according to their metabolic profile [11]. Therefore, we aim to get more insights about the individual effects of the diet depending on the specific metabolic characteristics of the study participants as a more innovative and personalized approach in the analysis of dietary exposures on FLI. Recent evidence suggests that a diet low in carbohydrates may be a promising therapeutic strategy in MAFLD; its effectiveness has been shown in intervention studies in obese subjects [12]. Accordingly, the replacement of carbohydrates with other energy-providing nutrients will be explored as well using energy substitution models.

## Materials and methods

### Study sample

The KORA (Cooperative Health Research in the Region of Augsburg) is a prospective population-based study of adults in the Region of Augsburg, Germany, and aims to examine the effects of environmental and lifestyle factors on non-communicable diseases and to contribute novel strategies for primary prevention [13]. From 1984 to 1995 the WHO firstly carried out the MONICA (Monitoring Trends and Determinants in Cardiovascular Disease) project which was then continued as the KORA study in 1996 by Helmholtz Center Munich [13]. From 1984 to 2001 four cross-sectional (S1 to S4) baseline surveys with around 18,000 randomly selected participants were conducted [14]. The KORA S4 survey was carried out from 1999 to 2001 and consisted of 4,261 inhabitants of the study region (city of Augsburg and two surrounding counties) aged 25 to 74 years [14]. In the follow-up study KORA Fit, 3,059 KORA participants born between 1945 and 1964 of each baseline survey were included; the study was performed in 2018 and 2019 [14]. Our study sample includes a total of 689 subjects (304 men and 385 women) of the S4 part of KORA-Fit who had available dietary intake data and blood serum measurements for the calculation of the fatty liver index and the estimation of the metabotypes. Ethical release was provided by the Ethics Committee of the Bavarian Medical Association (Bayerische Landesärztekammer). All study participants gave informed consent, and the study was conducted in accordance with the Declaration of Helsinki [13].

### Measurements of exposures

Body weight and height were measured in light clothing and without shoes; Body Mass Index (BMI) was calculated by the division of body weight (in kg) through the square of the height (in m) [15]. For the examination of waist circumference the measuring tape was placed between the distance of the lower rib margin and iliac crest [16]. Sociodemographic and lifestyle assessments were performed in computer assisted face-to-face interviews by specifically trained and certified medical personnel. Smoking was categorized as current, former or never; education status was grouped as < 12 years and 12 years or more of scholar education; physical activity was classified into categories subdivided by the estimated time per week spent on sports activities during leisure time in summer and winter. Furthermore, information about medication use and relevant medical diagnoses such as diabetes or hypertension was gathered. Participants who previously got medically diagnosed with diabetes and/or were under antidiabetic medication were classified as having diabetes. If the subjects baseline blood pressure exceeded values of systolic >  = 140 or diastolic >  = /90 mmHg, or receiving antihypertensive treatment, provided the subject is aware of hypertension, they were categorized as hypertensives [17].

Dietary data was collected via repeated 24-h food lists (24HFL) and a food frequency questionnaire (FFQ) with 246 and 148 items, respectively. The 24HFL was developed for the German National Cohort [18] and subjects were only asked to report type and frequency of food intake of the past day. The FFQ was based on the German version of the multilingual European Food Propensity Questionnaire which aims to determine dietary habits over the past 12 months [19]. The calculation of a person’s dietary intake in KORA Fit is based on the estimation of consumption probability and consumption amount. Details have been published previously [20]; briefly, consumption probability is determined for each food item for each individual based on three (at least two) 24HFLs and FFQ, while usual portion size for each item is estimated based on data from the Bavarian Food Consumption Survey II (BVS II) [20]. Consumption probabilities were estimated using logistic mixed models, adjusting for covariates and the FFQ data on consumption frequency. By means of mixed linear models, the consumption amount of each food item was modeled, adjusted for age, sex, BMI, smoking, physical activity, and education level. Consumption probability multiplied by consumption amount then results in the usual intake of each food item on any given day. Food items were then categorized into 17 food groups and 21 subgroups according to the EPIC-Soft classification scheme [21].

For our study we utilized data on the consumption of total fruits, total vegetables, total nuts, total meat with the additional subgroups beef, pork and poultry, total fish, total eggs, dairy products, whole grain, and soft drinks. Furthermore, to get more insights about specific dietary patterns of the study participants, the Alternate Healthy Eating Index (AHEI) and the Mediterranean Diet Score (MDS) were calculated as previously described [22, 23] and included in our analysis. The German food composition data base (BLS, version 3.02) was linked to the individual food items to enable the estimation of the average daily intake of energy-providing macronutrients, i.e. total fat, saturated fatty acids (SFA), monounsaturated fatty acids (MUFA), polyunsaturated fatty acids (PUFA), protein, carbohydrates and alcohol. Daily carbohydrate, total fat, fatty acid subgroup, protein, and alcohol intake was transformed as % of total energy intake by assuming a mean energy value of 9 kcal/g for total fat and fatty acid subgroups, 4 kcal/g for carbohydrates and protein and 7 kcal/g for alcohol.

While the analysis of liver biopsies is still described as the gold standard for fatty liver diagnosis, the FLI is a far more practical tool for population-based studies because of lower costs and its non-invasiveness [24]. The formula of Bedogni et al. was utilized to determine FLI values [10]:

$$FLI=\left({e}^{0.953\times {\text{log}}\left(triglycerides\right)+0.139\times BMI+0.718\times {\text{log}}\left(GGT\right)+0.053\times waist circumference-15.745}\right)\div \left(1+{e}^{0.953\times {\text{log}}\left(triglycerides\right)+0.139\times BMI+0.718\times {\text{log}}\left(GGT\right)+0.053\times waist circumference-15.745}\right)\times 100$$

The formula includes measured data of waist circumference, body mass index (BMI), serum triglycerides, and gamma glutamyl transferase (GGT). The FLI ranges from 0 to 100 with high numbers indicating a high probability of the presence of fatty liver [10]. According to Bedogni et al., FLI values < 30 seem to accurately exclude the presence of fatty liver while values > 60 indicate the presence of fatty liver [10]. However, FLI values between 30 and 60 appear to be not as accurate in the prediction of fatty liver and seem to be affected by variables such as age and sex due to recent research [25]. Thus in our study, we categorized the participants into three different FLI subgroups (< 30, normal; 30–60, indeterminate; > 60, severely increased).

Metabotypes were calculated by using a K-means clustering algorithm as described previously by Riedl et al. [26, 27]. For the creation of three different homogenous metabotype clusters we used an optimized set of parameters previously established by Dahal et al.; this set included measurements of BMI, serum triglycerides, uric acid, fasting glucose, high-density-lipoprotein cholesterol (HDLc), and Non-HDLc which was defined as the difference between total cholesterol and HDLc [28]. Regarding the three derived metabotype clusters, cluster 1 was the “healthy” metabotype, cluster 2 the “intermediate” and cluster 3 the most “unfavourable” metabotype [28]. All laboratory measurements were performed by the analysis of serum samples in the central laboratory Institute of Laboratory Medicine, LMU Munich, with a cobas c702 clinical chemistry analyser (Roche Diagnostics, Rotkreuz, Switzerland).

### Statistical analysis

Age, BMI, waist circumference, energy intake, alcohol consumption, FLI and all laboratory and dietary parameters were used as continuous variables. Due to their non-normally distributed characteristics regarding the results from the Shapiro–Wilk-test, all continuous parameters were described by medians and 25th-75th percentile range. To analyse differences between the three FLI subgroups, the Kruskal–Wallis-test was used and for the differences between males and females the Mann–Whitney-U-test was applied. Sex, education status, physical activity, smoking status, and the diagnosis of hypertension or diabetes were defined as categorial variables. To investigate significant differences between FLI subgroups, and for differences between males and females, the Chi-squared test (categorial variables) was used.

For the analysis of the associations of food items with FLI stratified by the metabotype subgroups, multivariable linear regression models were fitted for each food item. Multicollinearity and autocorrelation were tested using the variance inflation factor and Durbin Watson test, respectively. Homoscedasticity and the normal distribution of residuals were visually assessed using the scatterplot (predicted values vs. residuals) and the Q-Q-plot, respectively. The linearity assumption between each continuous covariate and the outcome was tested by the second-degree polynomial approach. Extreme outliers were removed with regard to the Cook’s D. Furthermore, we tested for interaction effects between sex and metabotypes for each food item using the Wald test. The Benjamini–Hochberg False-Discovery-Rate (FDR) method was used to additionally adjust the *p*-values of each model for multiple testing. Due to a low number of subjects in the metabotype 3 group, we summarized metabotypes 2 and 3 into one subgroup to get more robust results. All models were adjusted for age, sex, physical activity, education years, smoking status and total energy intake.

To assess the association of FLI as outcome with an isocaloric replacement of carbohydrates by fat subtypes (or total fat) and protein as exposure, a substitution model based on linear regression was calculated. The substitution model included total energy intake and all energy-providing nutrients (i.e., SFA, MUFA, PUFA (or total fat), protein and alcohol) except for carbohydrates and was additionally adjusted for potential confounding covariables. Variables for carbohydrates, fat subgroups (or total fat), protein and alcohol were adequately scaled so that the β-coefficients for energy-providing nutrients denote a change per 5% of total energy intake (5 E%). Thus, the estimated coefficient for, e.g., PUFA intake can be interpreted as the association of FLI with a 5E% increase in PUFA at the expense of carbohydrates while energy supply from other sources remains unchanged. The models were adjusted for age, sex, physical activity, education years, smoking status and total energy intake, and tested for all necessary model assumptions.

A *p*-value < 0.05 was defined as statistically significant. All analyses were performed with the R-software (R version 4.3.1).

## Results

### Study sample characteristics

Table 1 shows the characteristics of all study participants and stratified FLI subgroup. Our study sample of 689 persons (304 men and 385 women) is a subsample of the S4 part of KORA-Fit for which we could assess sufficient dietary information. Over the follow-up of the S4 cohort, deviation from a random sample of the population may have increased. The average age was 63 years and the participants had a mean BMI of 27.4 kg/m^2^. About 12% were current smokers, and 15% reported no physical activity.

**Table 1:** Characteristics of all study participants and by FLI

	**Total**		**Normal FLI**		**Indeterminate FLI**		**Severely increased FLI**		
	***n* = 689**		***n* = 215**		**n = 145**		***n* = 329**		
**Characteristics**	**Median (25th—75th percentile)**								***p*-value**
Age [years]	63	(58; 68)	61	(57; 67)	64	(59; 68)	64	(59; 68)	***0.001***
BMI [kg/m^2^]	27.4	(24.1; 30.7)	23.2	(21.7; 24.9)	25.8	(24.7; 27.8)	30.9	(28.7; 33.5)	** < *0.001***
Waist circumference [cm]	93.6	(82.8; 103.0)	78.2	(73.8; 84.0)	89.5	(85.0; 94.1)	103.5	(98.0; 109.5)	** < *0.001***
HDL [mg/dl]	63.8	(51.0; 77.0)	77.0	(66.5; 90.8)	68.0	(57.1; 78.0)	53.0	(45.0; 64.0)	** < *0.001***
non-HDL [mg/dl]	146.0	(121.0; 172.2)	135.9	(112.1; 158.1)	145.1	(121.0; 177.0)	152.0	(124.0; 181.0)	** < *0.001***
Triglycerides [mg/dl]	106.0	(77.0; 145.0)	74.8	(61.0; 94.0)	100.2	(76.2; 129.0)	136.0	(107.0; 179.0)	** < *0.001***
gGT [U/l]	24.0	(17.0; 37.0)	16.0	(13.0; 21.0)	24.0	(17.0; 32.0)	32.0	(23.0; 49.0)	** < *0.001***
AST [U/l]	24.0	(20.0; 28.0)	22.0	(19.0; 25.8)	24.1	(21.0; 27.6)	25.0	(21.0; 30.2)	** < *0.001***
ALT [U/l]	24.0	(18.7; 31.0)	18.9	(16.0; 24.0)	23.4	(19.8; 29.0)	29.0	(23.0; 37.0)	** < *0.001***
Uric acid [mg/dl]	5.2	(4.4; 6.3)	4.5	(3.9; 5.1)	5.3	(4.4; 5.9)	6.0	(5.0; 7.1)	** < *0.001***
Fasting glucose [mg/dl]	97	(92; 105)	93	(88; 97)	96	(92; 102)	103	(96; 112)	** < *0.001***
FLI	54.9	(24.4; 89.4)	15.3	(9.4; 22.9)	41.9	(35.3; 49.5)	90.5	(78.2; 100.2)	** < *0.001***
	**n (%)**								
Sex									
Male	304	(44.1)	46	(21.4)	67	(46.2)	191	(58.1)	** < *0.001***
Female	385	(55.9)	169	(78.6)	78	(53.8)	138	(41.9)	
Education [years]									
< = 12 years	417	(60.5)	114	(53.0)	82	(56.6)	221	(67.2)	***0.002***
> 12 years	272	(39.5)	101	(47.0)	63	(43.4)	108	(32.8)	
Physical activity									
> = 2 h/week	266	(38.6)	107	(49.8)	61	(42.1)	98	(29.8)	** < *0.001***
1 h/week	230	(33.4)	65	(30.2)	53	(36.6)	112	(34.0)	
< 1 h/week	88	(12.8)	20	(9.3)	10	(6.9)	58	(17.6)	
(almost) no activity	105	(15.2)	23	(10.7)	21	(14.5)	61	(18.5)	
Smoking									
Current smoker	82	(11.9)	21	(9.8)	15	(10.3)	46	(14.0)	0.139
Former smoker	298	(43.3)	91	(42.3)	56	(38.6)	151	(45.9)	
Never smoker	309	(44.8)	103	(47.9)	74	(51.0)	132	(40.1)	
Hypertension									
Yes	313	(45.4)	45	(20.9)	54	(37.2)	214	(65.0)	** < *0.001***
No	376	(54.6)	170	(79.1)	91	(62.8)	115	(35.0)	
Diabetes									
Yes	49	(7.1)	5	(2.3)	5	(3.4)	39	(11.9)	** < *0.001***
No	640	(92.9)	210	(97.7)	140	(96.6)	290	(88.1)	
Metabotype									
1	140	(20.3)	61	(28.4)	24	(16.6)	55	(16.7)	***0.001***
2	478	(69.4)	154	(71.6)	112	(77.2)	212	(64.4)	
3	71	(10.3)	(0.0)	(0.0)	9	(6.2)	62	(18.8)	

A total of 215 participants were categorized into the normal FLI subgroup, 145 subjects fell into the indeterminate FLI subgroup and 329 were attributed to the severely increased FLI subgroup. For sex, age, BMI, waist circumference, all tested laboratory parameters, FLI, education status, physical activity, hypertension and diabetes status, and metabotype significant differences between the FLI subgroups were observed. On average, participants that fell into the increased FLI subgroups were older, had more unfavourable anthropometric measurement results such as an increased BMI and waist circumference, as well as increased values for all laboratory parameters, with the exception of lower values for HDL. Moreover, participants with an increased FLI were more often male, had a lower educational status, were less physically active, were attributed to a more unfavourable metabotype, and were more frequently diagnosed with hypertension and diabetes.

### Habitual dietary consumption values

Tables 2 and 3 display the food and nutrient consumption values (in g per day) in all participants and stratified by FLI subgroups. Significant differences between subgroups were found for total vegetables, total nuts, dairy, total meat and the sub-groups beef, pork and poultry, for total fish, total eggs, soft drinks, whole grains, AHEI, MDS, energy (Kilocalories), alcohol, total fat, SFA, MUFA and total protein consumption. While on average persons with increased FLI values revealed a higher consumption of total meat, meat sub-groups, total fish, total eggs, soft drinks, as well as total energy, alcohol, total fat, SFA, MUFA, and total protein intake, they eat less total vegetables, total nuts, dairy, and whole grains. Furthermore, study participants with higher FLI values tend to have lower values of the AHEI and MDS.

**Table 2:** Habitual food consumption data and dietary patterns in all participants and by FLI

	**Total**		**Normal FLI**		**Indeterminate FLI**		**Severely increased FLI**		
**Food item**	**Median (25th-75th percentile)**								***p*-value**
Total vegetables [g/d]	166.2	(136.5; 202.7)	185.6	(152.8; 218.9)	160.1	(133.3; 202)	156.2	(130.9; 191.4)	** < *0.001***
Total fruits [g/d]	149.6	(93.2; 216.2)	159.5	(106.2; 224.8)	141.4	(91.1; 212.8)	148.7	(87.9; 210.8)	0.068
Total nuts [g/d]	4.4	(2.6; 13)	6.1	(2.8; 18.8)	4.2	(2.9; 12.9)	3.9	(2.4; 11.2)	** < *0.001***
Dairy [g/d]	177.5	(121.3; 259.7)	205.8	(135.8; 288.7)	177.5	(132.4; 259.7)	162.6	(112.1; 229.6)	** < *0.001***
Total meat [g/d]	101.1	(74.5; 132.7)	75.8	(63; 101.1)	95.2	(73.6; 115.2)	119.9	(95.1; 152.6)	** < *0.001***
Beef [g/d]	8.1	(6.1; 10.7)	6.7	(5.6; 9.1)	7.7	(6.1; 9.9)	9.2	(6.8; 12.3)	** < *0.001***
Pork [g/d]	15.6	(10.7; 21.3)	10.7	(8.6; 15.9)	13.5	(10.7; 16.9)	18.0	(13.7; 25.8)	** < *0.001***
Poultry [g/d]	10.4	(9.2; 17)	9.5	(7.1; 13.8)	11.8	(9.2; 16.8)	12.9	(9.6; 17.7)	** < *0.001***
Total fish [g/d]	18.7	(12.6; 27.1)	17.4	(11.2; 25.6)	17.7	(12.9; 24.2)	19.9	(13.5; 30.5)	** < *0.001***
Total eggs [g/d]	16.0	(11.4; 22.7)	14.1	(10.5; 21)	15.1	(11.1; 21.2)	17.6	(13; 24.8)	** < *0.001***
Softdrinks [g/d]	5.3	(3.4; 13.3)	3.4	(2.5; 5.2)	4.8	(3.3; 10.7)	7.2	(5; 25.6)	** < *0.001***
Whole grains [g/d]	15.3	(7.4; 35.3)	24.6	(9.1; 40.5)	18.3	(7.3; 35.1)	11.5	(6.6; 26.4)	** < *0.001***
AHEI	44.3	(37.6; 51.2)	48.0	(41.3; 56.1)	45.7	(39.3; 50.6)	41.4	(35.2; 48.1)	** < *0.001***
MDS	4	(3; 6)	5	(4; 6)	4	(3; 6)	4	(3; 5)	***0.001***

**Table 3:** Habitual nutrient consumption data in all participants and by FLI

	**Total**		**Normal FLI**		**Indeterminate FLI**		**Severely increased FLI**		
**Nutrient**	**Median (25th-75th percentile)**								***p*-value**
Energy (Kilocalories)									
[kcal/d]	1735.1	(1488.9; 2064.1)	1674.4	(1482.8; 2040.2)	1729.2	(1477.3; 2053.3)	1794.7	(1495.7; 2087.1)	***0.013***
Alcohol									
[g/d]	5.0	(2.3; 13.3)	4.3	(2.1; 8.6)	5.9	(3.0; 18.2)	5.6	(2.2; 16.6)	***0.024***
[%E]	2.0	(0.9; 5.4)	1.8	(0.9; 3.6)	2.4	(1.2; 7.4)	2.2	(0.8; 6.5)	
Total fat									
[g/d]	75.3	(65.0; 88.1)	72.0	(64.2; 87.2)	74.3	(63.4; 85.3)	77.3	(66.0; 90.0)	***0.021***
[%E]	39.1	(33.7; 45.7)	38.7	(34.5; 46.9)	38.7	(33.0; 44.4)	38.8	(33.0; 45.1)	
SFA									
[g/d]	33.1	(28.7; 38.6)	32	(28.1; 37.4)	32.5	(28.0; 37.9)	34.5	(29.5; 39.6)	***0.026***
[%E]	17.2	(14.9; 20.0)	17.2	(15.1; 20.1)	16.9	(14.6; 19.7)	17.3	(14.8; 19.9)	
MUFA									
[g/d]	26.9	(22.9; 32.3)	25.6	(22.5; 31.5)	26.8	(22.5; 30.8)	28.0	(23.9; 32.9)	***0.008***
[%E]	14.0	(11.9; 16.8)	13.8	(12.1; 16.9)	13.9	(11.7; 16.0)	14.0	(12.0; 16.5)	
PUFA									
[g/d]	9.7	(8.1; 11.9)	9.6	(7.9; 12.4)	9.3	(7.9; 11.5)	10.0	(8.5; 11.8)	0.071
[%E]	5.0	(4.2; 6.2)	5.2	(4.2; 6.7)	4.8	(4.1; 6.0)	5.0	(4.3; 5.9)	
Total protein									
[g/d]	66.9	(57.3; 77.4)	63.6	(55.1; 76.3)	66.9	(54.8; 75.1)	69.4	(60.1; 79.0)	** < *0.001***
[%E]	15.4	(13.2; 17.8)	15.2	(13.2; 18.2)	15.5	(12.7; 17.4)	15.5	(13.5; 17.6)	
Total carbohydrate									
[g/d]	180.6	(149.8; 214.9)	180.4	(150.6; 220.8)	179.8	(147.0; 213.0)	181.1	(149.8; 210.7)	0.873
[%E]	41.6	(34.5; 49.5)	43.1	(36.0; 52.7)	41.6	(34.0; 49.3)	40.4	(33.4; 47.0)	

Descriptive statistics stratified by sex are available in the supplementary data (Tables S1 and S2).

### Associations of diet with the FLI, stratified by metabotype clusters

The associations of the analysed food groups and sub-groups with FLI were shown in Table 4. Regarding all participants, significant positive associations were observed for total meat and the sub-groups beef, pork, and poultry, for total fish, total eggs, and soft drinks whereas negative associations were obtained for nuts, whole grain products, and the diet quality indices, AHEI and MDS. No associations existed between fruits, vegetables and dairy products consumption and FLI.

**Table 4:** Associations of food groups and dietary patterns with FLI, overall and stratified by metabotype cluster^a^

Food items	ß-estimate	95% CI	*p*-value	Adjusted *p*-value**
Total participants (*n* = 689)				
Total fruits [g/d]	0.006	(-0.026; 0.037)	0.715	0.739
Total vegetables [g/d]	-0.028	(-0.079; 0.022)	0.271	0.316
Total nuts [g/d]	-0.221	(-0.42; -0.022)	***0.029***	***0.041***
Total meat [g/d]	0.466	(0.389; 0.543)	** < *0.001***	** < *0.001***
Beef [g/d]	0.801	(0.229; 1.372)	***0.006***	***0.011***
Pork [g/d]	1.016	(0.694; 1.338)	** < *0.001***	** < *0.001***
Poultry [g/d]	0.557	(0.271; 0.842)	** < *0.001***	** < *0.001***
Total fish [g/d]	0.325	(0.173; 0.478)	** < *0.001***	** < *0.001***
Total eggs [g/d]	0.607	(0.42; 0.794)	** < *0.001***	** < *0.001***
Total dairy [g/d]	-0.004	(-0.029; 0.021)	0.739	0.739
Whole grains [g/d]	-0.221	(-0.347; -0.095)	***0.001***	***0.001***
Softdrinks [g/d]	0.024	(0.002; 0.045)	***0.033***	***0.041***
AHEI	-0.908	(-1.163; -0.654)	** < *0.001***	** < *0.001***
MDS	-1.913	(-3.353; -0.473)	***0.009***	***0.014***
**Food items**	**ß-estimate**	**95% CI**	***p*-value**	Adjusted *p*-value**
Metabotype 1 (*n* = 140)				
Total fruits [g/d]	-0.008	(-0.074; 0.057)	0.805	0.835
Total vegetables [g/d]	-0.041	(-0.161; 0.079)	0.504	0.672
Total nuts [g/d]	0.032	(-0.328; 0.392)	0.861	0.861
Total meat [g/d]	0.273	(0.081; 0.464)	***0.006***	***0.016***
Beef [g/d]	0.207	(-0.801; 1.215)	0.685	0.781
Pork [g/d]	0.605	(-0.269; 1.479)	0.173	0.276
Poultry [g/d]	0.884	(0.103; 1.665)	***0.027***	0.058
Total fish [g/d]	0.359	(-0.029; 0.746)	0.069	0.129
Total eggs [g/d]	0.538	(0.076; 1)	***0.023***	0.053
Total dairy [g/d]	0.012	(-0.033; 0.057)	0.591	0.719
Whole grains [g/d]	-0.206	(-0.51; 0.098)	0.182	0.276
Softdrinks [g/d]	0.03	(-0.015; 0.075)	0.187	0.276
AHEI	-0.505	(-1.114; 0.103)	0.103	0.18
MDS	-1.069	(-4.678; 2.541)	0.559	0.711
**Food items**	**ß-estimate**	**95% CI**	***p*-value**	Adjusted *p*-value**
Metabotypes 2 and 3 (summarized) (*n* = 549)				
Total fruits [g/d]	0.006	(-0.029; 0.042)	0.725	0.781
Total vegetables [g/d]	-0.021	(-0.076; 0.034)	0.457	0.64
Total nuts [g/d]	-0.32	(-0.554; -0.085)	***0.008***	***0.019***
Total meat [g/d]	0.487	(0.404; 0.571)	** < *0.001***	** < *0.001***
Beef [g/d]	1.002	(0.322; 1.682)	***0.004***	***0.012***
Pork [g/d]	1.083	(0.734; 1.433)	** < *0.001***	** < *0.001***
Poultry [g/d]	0.516	(0.208; 0.824)	***0.001***	***0.004***
Total fish [g/d]	0.3	(0.136; 0.465)	** < *0.001***	***0.002***
Total eggs [g/d]	0.629	(0.424; 0.834)	** < *0.001***	** < *0.001***
Total dairy [g/d]	-0.006	(-0.036; 0.024)	0.711	0.781
Whole grains [g/d]	-0.234	(-0.371; -0.097)	***0.001***	***0.004***
Softdrinks [g/d]	0.026	(0; 0.051)	***0.046***	0.091
AHEI	-0.987	(-1.264; -0.709)	** < *0.001***	** < *0.001***
MDS	-2.352	(-3.911; -0.792)	***0.003***	***0.011***

^a^linear regression models adjusted for sex, age, physical activity, education years, smoking status, energy intake, and metabotype cluster. CI, confidence interval

^**^ False discovery rate (FDR)-adjusted

For most food parameters, with the exception for total vegetables and the AHEI, a significant interaction with metabotypes (food * metabotype) was observed. These findings were supported by the results of the stratified analysis. For participants in the metabotype 1 cluster, the consumption of total meat, poultry and total eggs was significantly associated with the FLI, and only total meat remained its significance after correction for multiple testing. For the summarized metabotype cluster 2 and 3, we recognized many more significant associations which were similar to those reported for all participants; in the summarized metabotype cluster of 2 and 3, the consumption of total meat and the sub-groups beef, pork, and poultry, for total fish, total eggs, and softdrinks showed positive associations with FLI whereas negative associations were obtained for nuts, whole grain products, AHEI and MDS. However, the observed association with softdrinks lost its significance after FDR adjustment. Moreover, the ß-estimates were higher also in the metabotype 2 + 3 groups as compared to all study participants, suggesting greater effects of dietary intakes on FLI in this subgroup. The separate findings for cluster 2 and 3 are displayed in the supplementary data (Table S3).

### Effects of substituting carbohydrates with other macronutrients on the FLI

Table 5 provides the results of the carbohydrate substitution model. Replacing total carbohydrates with an isoenergetic amount of MUFA and protein was significantly positively associated with FLI. A greater effect was shown for the replacement of carbohydrates with protein which increased the FLI on average by 26.121 points compared to MUFA which increased the FLI by 11.482 points per 5 energy-% increase. On the other hand, an increase in PUFA intake at the expense of an isoenergetic amount of carbohydrate intake was significantly related with reduced FLI values by -23.332 points. Other energy substitution models (Table S4) confirm these findings.

**Table 5:** Effects of substitution of carbohydrates by macronutrients (per 5 energy percent) on Fatty Liver Index^a^

Per 5 energy-% increase	ß-estimate	95% CI	*p*-value
SFA	-6.324	(-14.391; 1.744)	0.124
MUFA	11.482	(1.129; 21.836)	***0.03***
PUFA	-23.332	(-40.003; -6.662)	***0.006***
Protein	26.121	(18.825; 33.416)	** < *0.001***
Alcohol	0.775	(-3.384; 4.934)	0.715

^a^Substitution models contained total energy intake, SFA, MUFA, PUFA, protein, and alcohol intake. Estimates are therefore interpreted as the association of FLI with a 5 E% increase in e.g. PUFA at the expense of carbohydrates while energy supply from other macronutrients remains unchanged; linear regression models adjusted for sex, age, physical activity, education years, smoking status and energy intake. CI, confidence interval

## Discussion

### Main findings

In our study we observed beneficial effects of plant-based food items and dietary patterns, especially nuts, whole grain products, the AHEI, and the MDS on FLI values in contrast to unfavourable effects of soft drinks and animal-derived products such as meat, eggs or fish. According to the results of the energy substitution models, a higher PUFA intake exerts a favourable relationship with FLI while protein and MUFA intakes are associated with higher FLI. Regarding metabotype subgroups, participants with metabotype 2 and 3 revealed stronger associations with dietary factors, as compared to the metabolically more healthy participants in cluster 1. These findings argue for a differential benefit from dietary modifications between metabotypes.

### Effects of foods of plant or animal origin on fatty liver index

The positive outcomes of plant-based diets and food items regarding fatty liver are concordant with previously conducted studies investigating the effects of diet on specific liver markers or liver imaging diagnostics. In 2023, a cohort study from North China with 14,541 participants reported a significant risk reduction for MAFLD measured by liver ultrasonography when replacing animal derived food items, i.e. meat, eggs or fish, with an equivalent serving of whole grains [29]. In the 2005–2018 NHANES study with 25,360 adult participants from the US, especially nut intake was significantly associated with lower FLI values [30]. Whole grain products and nuts are both rich sources of dietary fiber, minerals and antioxidants which are known to lower the risk of chronic diseases [31, 32], and especially fiber has been shown to yield a protective effect on the development of MAFLD [33]. Whole grains and nuts are characteristic components of the Mediterranean Diet which also has been suggested as an effective dietary approach in the management of MAFLD [34, 35]; also in our study, a high MDS was inversely associated with FLI.

High consumption of animal-derived food items, especially meat products, has been shown to detrimentally affect liver health [36]. A meta-analysis from 2020 of 24 observational studies found that red meat consumption is associated with a higher likelihood of MAFLD but did not report unfavourable effects of other animal products, such as dairy, eggs or fish [37]. Especially the presence of heme–iron in red meat which has also been shown to unfavourably affect liver health [38] could be a possible explanation for this observation. While other studies reported conflicting results for egg consumption [39, 40], regarding fish and dairy products even inverse relationships with fatty liver measurements were reported; these findings may be attributable to the content of long-chain omega-3-fatty acids in fish or probiotics and minerals like calcium present in dairy products [41, 42]. These reports are partly in contrast to the findings of our study which showed unfavourable associations for all animal-based food items, except for dairy products. A possible explanation could be the low amount and differences in the processing of such foods. The last report from the 2023 European Market Observatory for Fisheries and Aquaculture products (EUMOFA) described the majority of the fish in Germany were sold in a processed form (e.g. preserved in brine, salt water or with a sauce) [43], possibly outweighing health-favourable aspects of sea food, especially long-chain n3-PUFA. Processed fish consumption has already been associated with a higher all-cause mortality risk in previous studies [44].

We also found a positive association between soft drink consumption and FLI values, which may be due to their high sugar, and especially high fructose content [45]. This observation is in concordance with the previously mentioned meta-analysis which came to the same conclusion [37]. Fructose is known as a main mediator in the development of fatty liver [8] and clinical guidelines from the European Society for Clinical Nutrition and Metabolism (ESPEN) generally advise to limit its consumption [35].

### Effects of different macronutrients on fatty liver

While the beneficial effects and mechanisms of the isocaloric replacement of dietary carbohydrates with total fat on liver fat content has been demonstrated in intervention studies in obese subjects (recently reviewed by Lundsgaard et al. [46]), less is known about the impact of the different types of fatty acids and the related food sources.

A potential mechanism of the liver health-promoting effects of plant-derived foods, especially nuts and seeds, is their high PUFA content. In randomized controlled trials, PUFAs have been shown to improve metabolic health by lowering liver fat accumulation compared to an isocaloric amount of SFAs [47, 48]. Conversely, the effects of protein and MUFA consumption on the development of fatty liver are still inconclusive. Interventional studies that directly assessed the effects of MUFAs or protein reported beneficial outcomes on indicators of fatty liver [49–51] while observational studies did not find any or even unfavourable associations [52, 53]. Moreover, the dietary source seems to play a major role in the effects of both macronutrients on liver health which could explain the diverging findings in the mentioned studies. Proteins from plant sources have been shown to have beneficial effects on MAFLD risk while the opposite seems to be the case for animal-based protein [29]. Also, MUFA administration in interventional studies is often derived from plant products such as olive oil [49]. In contrast, the main sources of protein and MUFAs in countries like Germany are typically animal products, especially meat [54, 55], which may also explain the observed associations of protein and MUFA consumption with FLI values in our study. Surprisingly, for SFA intake we did not observe a significant association with FLI which may be due to its presence (partly as medium-chain SFA) in dairy products which are a main contributor to total SFA consumption in Europe [55]. Moreover, recent evidence suggests that the likelihood of the development of MAFLD is indeed inversely associated with dairy consumption [42].

However, if consumed in excess, dietary fat appears to promote overall energy consumption and thus, contribute to the development of metabolic diseases including obesity [56]. Potential mechanisms for this observation include a detrimental modulation of hormones such as ghrelin, glucagon-like peptide-1, and cholecystokinin (involved in the regulation of appetite) as well as a potential dysregulation of gastrointestinal motor function which has been comprehensively reviewed by Little et al. [56]. The importance of gut hormones on MAFLD development is also supported by evidence from clinical intervention studies involving bariatric surgery which, besides effectively promoting long-term body weight reduction, also could ameliorate obesity-related MAFLD by the modulation of hormones associated with appetite regulation [57]. Therefore, dietary fat could be consumed in exchange, and not on top of dietary carbohydrates to avoid potential detrimental effects on gut hormones and excessive energy intake.

### Influence of metabotypes on the relationship between diet and MAFLD

Besides the higher prevalence of MAFLD in obese populations, it has been shown that obese subjects with MAFLD additionally show a more unfavourable metabolic profile compared to lean MAFLD patients [58]. Therefore, the individual metabotype (as used here) may be a potential effect modifier by influencing the individual response to environmental factors such as diet. In fact, in our study we got clear indication of effect modification by metabotype clusters for the association of dietary factors and the prevalence of FLI.

Stratifying study participants into metabotype subgroups is an emerging approach in nutrition research to identify groups that benefit most from specific dietary modifications [11]. Previous studies investigated the effects of food items or nutrients on specific disease markers in different metabotype subgroups. For example, it has been shown that the effect of bread consumption on postprandial insulin response [59] or the influence of vitamin D on markers of the metabolic syndrome [60] differ depending on the individual metabotype. However, studies with more detailed dietary data to analyse the associations of several food groups on health outcomes while also considering effect modification by metabotypes are still rare. In an earlier study, Riedl et al. used metabotype subgrouping to investigate the effects of the consumption of different food items on type 2 diabetes mellitus (T2DM) [27]. Three metabotype clusters were created which were based on BMI and 15 other parameters derived from blood serum measurements. Cluster 1 was defined as the more favourable metabolic profile while cluster 3 was described as the most unfavourable. While in cluster 1 significant positive associations with T2DM were reported only for increased intakes of total meat and processed meat, in the cluster 3, however, a significant positive association with T2DM was found for the increased consumption of sugar-sweetened beverages and an inverse association was observed for increased fruit intake. The authors concluded that depending on the individual metabotype, dietary factors may exert differential effects on the risk of T2DM [27]. Our study complements these results by using a similar metabotyping approach in the analysis of the consumption of different food items with the FLI. We observed significant associations of meat consumption with the FLI in all metabotype subgroups which is in line with the clear negative impact of meat intake on fatty liver as reported frequently in previous studies [36, 37]. The conflicting results for other food items in the literature, especially of foods of animal origin, may be partly explained by possible heterogenous metabolic profiles of the subjects included in the studies, a point which should be addressed in further investigations.

### Practical implications and potential applications

The knowledge of the differential associations between dietary factors and fatty liver depending on the individuals’ metabotype may contribute to a more personalized approach in dietary counselling. In 2015, a study by O’Donovan et al. firstly suggested a targeted method based on individual metabotypes to individualize dietary advices in clinical settings [61] which could be expanded on a population-based level to provide innovative primary prevention strategies for public health. In fact, Hillesheim et al. could demonstrate the effectiveness of such a concept in a randomized trial [62].

In the study by Riedl et al., the authors also suggested that the prevention of diabetes could especially benefit from a change in the dietary behaviour of individuals with an unfavourable metabotype [27]. Thus, this implication may be also applicable to the primary prevention of NAFLD according to the findings of our study where we so more distinct relationships between dietary factors on FLI in clusters 2 and 3. Explicitly, the results of our study may suggest a reduction in the consumption of animal-based food items and soft drinks in favour of PUFA-rich foods, nuts as well as whole grain products in subjects with a more unfavourable metabolic profile as a working hypothesis for primary prevention of MAFLD. Moreover, for those individuals, it may be also advisable to follow a healthy dietary pattern as indicated by the AHEI to further reduce the likelihood of fatty liver development. Besides the current ESPEN guidelines for NAFLD which mainly recommend adherence to an energy-reduced Mediterranean diet [35], the findings of our study may add new possibilities for the dietary prevention strategies of NAFLD by suggesting specific food item choices and respecting the individual metabolic profile. However, future, large, prospective studies and finally intervention studies are needed to confirm our results and the delineated hypotheses.

### Strengths and limitations

Strengths of our study are a food-based approach compared to previously nutrient-based analyses as a more practical method for generating public health recommendations, and the investigation on a population-based level with a high number of study subjects. Also, our study is among the first to stratify the analysis of food items and FLI by metabolic characteristics (i.e., metabotypes) of the study participants which may allow more individualized and effective dietary recommendations regarding fatty liver prevention. However, some limitations have to be addressed. Compared to metabotype cluster 2, cluster 1 and, especially cluster 3 contained remarkably fewer participants which led to a more uneven distribution of the study participants across the metabotype clusters. Thus, we combined the persons in clusters 2 and 3 for the statistical analysis, but the relatively small sample size of cluster 1 remains as a potential limitation. Another weakness of population-based studies is the measurement error adherent to dietary assessment data. As a consequence of recall bias, study participants may not be able to remember the exact food items and portion sizes which were required to precisely answer the questions about their habitual diet. However, with the combination of repeated 24-h recalls and an FFQ we tried to improve the validity and precision of the dietary intake data. Moreover, the derivation of macronutrient intake values is based on average values for the individual food items (as taken from the German food composition database), and the true macronutrient content in food actually consumed may deviate from the average. Also, the fatty liver index is not as precise as other methods to classify subjects according to their liver fat content (MR imaging; liver biopsy) and to classify fatty liver disease. Moreover, further data on other laboratory parameters for insulin resistance such as the HOMA index were not available in our dataset for a more precise description of the metabolic health of the study sample. Furthermore, the KORA S4 Fit survey was performed in a specific population and the results may not be fully applicable to other age or ethnic groups.

## Conclusion

In our population-based study, the consumption of nuts, whole grains, and PUFAs or following a healthy dietary pattern, represented as AHEI and MDS, was inversely associated with FLI, whereas for the intake of soft drinks and animal products such as meat, fish and eggs, we reported positive associations with FLI. The impact of diet on fatty liver may be modified by the individual metabotype of the study participants which needs further investigation in future prospective studies. By offering new insights about the interplay of diet and fatty liver at the population level, our study may promote the development of novel primary prevention strategies for MAFLD.

### Supplementary Information

**Additional file 1: Table S1. **Characteristics of all study participants and by sex. **Table S2.** Habitual food and nutrient consumption data and dietary patterns in all participants and by metabotype cluster. **Table S3.** Associations of food groups and subgroups and dietary patterns with FLI, overall and stratified by metabotype cluster. **Table S4.** Effects of substitution of macronutrients by saturated fatty acids (SFA), monounsaturated fatty acids (MUFA), polyunsaturated fatty acids (PUFA), protein and alcohol (per 5 energy percent) on Fatty Liver Index (FLI).

## Data Availability

The data are subject to national data protection laws, and restrictions were imposed by the Ethics Committee of the Bavarian Chamber of Physicians to ensure data privacy of the study participants. Therefore, data cannot be made freely available in a public repository. However, data can be requested through an individual project agreement with KORA via the online portal KORA (https://www.helmholtz-munich.de/en/epi. Accessed 06 Dec 2023).

## Abbreviations

24HFLs: 24-H food lists
FFQ: Food frequency questionnaire
KORA: Cooperative Health Research in the region of Augsburg
MDS: Mediterranean Diet Score
AHEI: Alternate Healthy Eating Index
MONICA: Monitoring trends and determinants in cardiovascular disease
WHO: World Health Organization
EPFQ: European Food Propensity Questionnaire
VIF: Variance-Inflation-Factor
BMI: Body-Mass-Index
FDR: False-Discovery-Rate
SFA: Saturated fatty acids
PUFA: Polyunsaturated fatty acids
MUFA: Monounsaturated fatty acids
ESPEN: European Society for Clinical Nutrition and Metabolism
