# Supplementary material for: Association of the habitual dietary intake with the fatty liver index and effect modification by metabotypes in the population-based KORA-Fit study
Source: Lipids Health Dis. 2024 Apr 4;23:99. doi: 10.1186/s12944-024-02094-0 (PMC10993479; doi:10.1186/s12944-024-02094-0)
Supplement: Supplementary file 1 — Additional file 1: Table S1. Characteristics of all study participants and by sex. Table S2. Habitual food and nutrient consumption data and dietary patterns in all participants and by metabotype cluster. Table S3. Associations of food groups and subgroups and dietary patterns with FLI, overall and stratified by metabotype cluster. Table S4. Effects of substitution of macronutrients by saturated fatty acids (SFA), monounsaturated fatty acids (MUFA), polyunsaturated fatty acids (PUFA), protein and alcohol (per 5 energy percent) on Fatty Liver Index (FLI). [file 12944_2024_2094_MOESM1_ESM.docx]

**Supplementary data for the article “Association of the habitual dietary intake with the fatty liver index and effect modification by metabotypes in the population-based KORA-Fit study”**

| **Table S1.** Characteristics of the study participants and by sex | | | | | | | |
| --- | --- | --- | --- | --- | --- | --- | --- |
|  | **Total** | | **Males** | | **Females** | |  |
|  | **n = 689** | | **n = 304** | | **n = 385** | |  |
| **Characteristics** | **Median (25th-75th percentile)** | | | | | | **p-value** |
| Age [years] | 63 | (58; 68) | 64 | (58; 68) | 63 | (59; 68) | 0.659 |
| BMI [kg/m²] | 27.4 | (24.1; 30.7) | 27.9 | (25.3; 31.1) | 26.64 | (23.4; 30.4) | ***0.001*** |
| Waist circumference [cm] | 93.6 | (82.8; 103.0) | 99.6 | (91.8; 107.9) | 86.5 | (78.1; 97.4) | ***< 0.001*** |
| FLI | 54.9 | (24.4; 89.4) | 79.1 | (41.4; 96.5) | 36.2 | (16.1; 76.9) | ***< 0.001*** |
| HDL [mg/dl] | 61.0 | (49.0; 75.4) | 53.0 | (43.5; 64.0) | 71.0 | (57.0; 83.5) | ***< 0.001*** |
| non-HDL [mg/dl] | 146.0 | (120.1; 174.0) | 144.3 | (115.0; 174.9) | 147.0 | (122.3; 173.9) | 0.200 |
| Triglycerides [mg/dl] | 107.0 | (77.0; 150.3) | 114.0 | (81.3; 166.0) | 100.0 | (73.6; 139.0) | ***< 0.001*** |
| gGT [U/l] | 25.0 | (17.0; 39.0) | 32.0 | (22.0; 48.0) | 20.0 | (14.0; 30.0) | ***< 0.001*** |
| AST [U/l] | 24.0 | (20.3; 28.1) | 26.0 | (22.0; 30.3) | 22.5 | (19.1; 26.7) | ***< 0.001*** |
| ALT [U/l] | 24.8 | (19.0; 32.0) | 29.0 | (23.0; 36.2) | 21.5 | (17.0; 27.0) | ***< 0.001*** |
| Uric acid [mg/dl] | 5.3 | (4.4; 6.4) | 6.28 | (5.4; 7.2) | 4.7 | (4.1; 5.4) | ***< 0.001*** |
| Fasting glucose [mg/dl] | 97.0 | (92; 105) | 101.0 | (94; 110) | 95.0 | (90; 102) | ***< 0.001*** |
|  | **n (%)** | | | | | |  |
| Education [years] |  |  |  |  |  |  |  |
| <=12 years | 417 | (60.5) | 166 | (54.6) | 251 | (65.2) | ***< 0.001*** |
| >12 years | 272 | (39.5) | 138 | (45.4) | 134 | (34.8) |  |
| Physical activity |  |  |  |  |  |  |  |
| >=2h / week | 266 | (38.6) | 127 | (41.8) | 139 | (36.1) | 0.402 |
| 1h / week | 230 | (33.4) | 95 | (31.3) | 135 | (35.1) |  |
| <1h / week | 88 | (12.8) | 40 | (13.2) | 48 | (12.5) |  |
| (almost) no activity | 105 | (15.2) | 42 | (13.8) | 63 | (16.4) |  |
| Smoking |  |  |  |  |  |  |  |
| Current smoker | 82 | (11.9) | 40 | (13.2) | 42 | (10.9) | ***< 0.001*** |
| Former smoker | 298 | (43.3) | 149 | (49.0) | 149 | (38.7) |  |
| Never smoker | 309 | (44.9) | 115 | (37.8) | 194 | (50.4) |  |
| Hypertension |  |  |  |  |  |  |  |
| Yes | 313 | (45.4) | 165 | (54.3) | 148 | (38.4) | ***< 0.001*** |
| No | 376 | (54.6) | 139 | (45.7) | 237 | (61.6) |  |
| Diabetes |  |  |  |  |  |  |  |
| Yes | 49 | (7.1) | 25 | (8.2) | 24 | (6.2) | 0.371 |
| No | 640 | (92.9) | 279 | (91.8) | 361 | (93.8) |  |
| Metabotype |  |  |  |  |  |  |  |
| 1 | 140 | (20.3) | 72 | (23.7) | 68 | (17.7) | ***< 0.001*** |
| 2 | 478 | (69.4) | 185 | (60.9) | 293 | (76.1) |  |
| 3 | 71 | (10.3) | 47 | (15.5) | 24 | (6.2) |  |

| **Table S2**. Habitual food and nutrient consumption data and dietary patterns in all participants and by metabotype cluster | | | | | | | |
| --- | --- | --- | --- | --- | --- | --- | --- |
|  | **Total** | | **Males** | | **Females** | |  |
| **Food item** | **Median (25th-75th percentile)** | | | | | | **p-value** |
| Total fruits [g/d] | 149.6 | (93.2; 216.2) | 141.9 | (79.3; 214.6) | 151.9 | (103.5; 216.3) | ***0.041*** |
| Total vegetables [g/d] | 166.2 | (136.5; 202.7) | 150.9 | (125.7; 183.8) | 180.8 | (149.9; 218.3) | ***< 0.001*** |
| Total nuts [g/d] | 4.4 | (2.6; 13.0) | 5.4 | (3.2; 14.0) | 3.8 | (2.4; 11.9) | ***< 0.001*** |
| Total meat [g/d] | 101.1 | (74.5; 132.7) | 133.9 | (111.4; 160.0) | 79.3 | (65.0; 96.2) | ***< 0.001*** |
| Beef [g/d] | 8.1 | (6.1; 10.7) | 10.2 | (8.8; 14.7) | 6.2 | (5.2; 7.8) | ***< 0.001*** |
| Pork [g/d] | 15.6 | (10.7; 21.3) | 20.8 | (16.3; 26.6) | 11 | (9.3; 15.3) | ***< 0.001*** |
| Poultry [g/d] | 10.4 | (9.2; 17) | 13.5 | (9.97; 23.1) | 9.5 | (7.1; 13) | ***< 0.001*** |
| Total fish [g/d] | 18.7 | (12.6; 27.1) | 19.7 | (14.2; 30.1) | 17.6 | (11.9; 25.6) | ***< 0.001*** |
| Total eggs [g/d] | 16.0 | (11.4; 22.7) | 16.0 | (12.1; 23.2) | 15.9 | (11.2; 22.3) | 0.371 |
| Dairy [g/d] | 177.5 | (121.3; 259.7) | 156.9 | (108.3; 237.4) | 193.5 | (133.5; 268.7) | ***< 0.001*** |
| Whole grains [g/d] | 15.3 | (7.4; 35.3) | 14.8 | (7.3; 33.5) | 15.8 | (7.6; 36.1) | 0.936 |
| Sugar sweetened beverages [g/d] | 5.3 | (3.4; 13.3) | 7.8 | (5.3; 21.7) | 3.8 | (2.8; 5.9) | ***< 0.001*** |
| AHEI | 44.3 | (37.6; 51.2) | 42.8 | (36.5; 48.9) | 45.6 | (38.8; 52.5) | ***< 0.001*** |
| MDS | 4 | (3; 6) | 5 | (3; 6) | 4 | (3; 6) | ***0.015*** |
| Energy (Kilocalories) [kcal/d] | 1735.1 | (1488.9; 2064.1) | 2051.1 | (1839.2; 2291.6) | 1533.8 | (1383.3; 1711.4) | ***< 0.001*** |
| Alcohol consumption [g/d] | 5.0 | (2.3; 13.3) | 13.3 | (6.3; 25.7) | 2.7 | (1.6; 5.2) | ***< 0.001*** |
| Total fat consumption [g/d] | 75.3 | (65.0; 88.1) | 86.4 | (76.6; 97.5) | 67.1 | (60.3; 76.2) | ***< 0.001*** |
| SFA consumption [g/d] | 33.1 | (28.7; 38.6) | 38.0 | (33.9; 42.6) | 30.2 | (26.9; 33.9) | ***< 0.001*** |
| MUFA consumption [g/d] | 26.9 | (22.9; 32.3) | 31.2 | (27.6; 36.0) | 24.0 | (21.0; 27.5) | ***< 0.001*** |
| PUFA consumption [g/d] | 9.7 | (8.1; 11.9) | 11.1 | (9.5; 13.4) | 8.6 | (7.5; 10.4) | ***< 0.001*** |
| Total protein consumption [g/d] | 66.9 | (57.3; 77.4) | 76.0 | (68.0; 87.2) | 59.5 | (53.2; 68.1) | ***< 0.001*** |
| Total carbohydrate consumption [g/d] | 180.6 | (149.8; 214.9) | 206.7 | (179.9; 240.8) | 159.7 | (139.7; 189.1) | ***< 0.001*** |

| **Table S3.** Associations of food groups and subgroups and dietary patterns with FLI, overall and stratified by metabotype cluster* | | | | |
| --- | --- | --- | --- | --- |
| **Food items** | **ß-estimate** | **95% CI** | **p-value** | **Adjusted p-value**** |
| Metabotype 1 (n = 140) | | | | |
| Total fruits [g/d] | -0.008 | (-0.074; 0.057) | 0.805 | 0.835 |
| Total vegetables [g/d] | -0.041 | (-0.161; 0.079) | 0.504 | 0.672 |
| Total nuts [g/d] | 0.032 | (-0.328; 0.392) | 0.861 | 0.861 |
| Total meat [g/d] | 0.273 | (0.081; 0.464) | ***0.006*** | ***0.016*** |
| Beef [g/d] | 0.207 | (-0.801; 1.215) | 0.685 | 0.781 |
| Pork [g/d] | 0.605 | (-0.269; 1.479) | 0.173 | 0.276 |
| Poultry [g/d] | 0.884 | (0.103; 1.665) | ***0.027*** | 0.058 |
| Total fish [g/d] | 0.359 | (-0.029; 0.746) | 0.069 | 0.129 |
| Total eggs [g/d] | 0.538 | (0.076; 1) | ***0.023*** | 0.053 |
| Total dairy [g/d] | 0.012 | (-0.033; 0.057) | 0.591 | 0.719 |
| Whole grains [g/d] | -0.206 | (-0.51; 0.098) | 0.182 | 0.276 |
| Softdrinks [g/d] | 0.030 | (-0.015; 0.075) | 0.187 | 0.276 |
| AHEI | -0.505 | (-1.114; 0.103) | 0.103 | 0.180 |
| MDS | -1.069 | (-4.678; 2.541) | 0.559 | 0.711 |
| **Food items** | **ß-estimate** | **95% CI** | **p-value** | **Adjusted p-value**** |
| Metabotype 2 (n = 478) | | | | |
| Total fruits [g/d] | 0.009 | (-0.028; 0.047) | 0.617 | 0.700 |
| Total vegetables [g/d] | -0.034 | (-0.092; 0.025) | 0.257 | 0.392 |
| Total nuts [g/d] | -0.312 | (-0.559; -0.064) | ***0.014*** | ***0.048*** |
| Total meat [g/d] | 0.527 | (0.438; 0.616) | ***<0.001*** | ***<0.001*** |
| Beef [g/d] | 1.092 | (0.382; 1.801) | ***0.003*** | ***0.016*** |
| Pork [g/d] | 1.295 | (0.902; 1.687) | ***<0.001*** | ***<0.001*** |
| Poultry [g/d] | 0.492 | (0.155; 0.829) | ***0.004*** | ***0.023*** |
| Total fish [g/d] | 0.282 | (0.108; 0.456) | ***0.002*** | ***0.011*** |
| Total eggs [g/d] | 0.595 | (0.368; 0.821) | ***<0.001*** | ***<0.001*** |
| Total dairy [g/d] | -0.013 | (-0.045; 0.019) | 0.433 | 0.593 |
| Whole grains [g/d] | -0.286 | (-0.432; -0.141) | ***<0.001*** | ***0.001*** |
| Softdrinks [g/d] | 0.040 | (0.009; 0.071) | ***0.013*** | ***0.048*** |
| AHEI | -1.011 | (-1.304; -0.717) | ***<0.001*** | ***<0.001*** |
| MDS | -2.369 | (-4.025; -0.712) | ***0.005*** | ***0.023*** |
| **Food items** | **ß-estimate** | **95% CI** | **p-value** | **Adjusted p-value**** |
| Metabotype 3 (n = 71) | | | | |
| Total fruits [g/d] | 0.027 | (-0.025; 0.079) | 0.306 | 0.442 |
| Total vegetables [g/d] | 0.043 | (-0.033; 0.119) | 0.261 | 0.392 |
| Total nuts [g/d] | -0.272 | (-0.56; 0.015) | 0.063 | 0.145 |
| Total meat [g/d] | 0.100 | (-0.006; 0.205) | 0.063 | 0.145 |
| Beef [g/d] | 0.326 | (-0.598; 1.251) | 0.482 | 0.632 |
| Pork [g/d] | -0.130 | (-0.463; 0.204) | 0.438 | 0.593 |
| Poultry [g/d] | 0.272 | (-0.031; 0.574) | 0.077 | 0.154 |
| Total fish [g/d] | 0.191 | (-0.014; 0.396) | 0.067 | 0.145 |
| Total eggs [g/d] | 0.214 | (-0.05; 0.479) | 0.110 | 0.201 |
| Total dairy [g/d] | 0.037 | (0.004; 0.07) | ***0.029*** | 0.080 |
| Whole grains [g/d] | 0.196 | (0.016; 0.377) | ***0.034*** | 0.088 |
| Softdrinks [g/d] | 0.006 | (-0.021; 0.032) | 0.673 | 0.719 |
| AHEI | -0.107 | (-0.491; 0.278) | 0.580 | 0.689 |
| MDS | -0.420 | (-2.434; 1.595) | 0.677 | 0.719 |
| * linear regression models adjusted for sex, age, physical activity, education years, smoking status, energy intake, and metabotype cluster. CI, confidence interval; ** False discovery rate (FDR)-adjusted | | | | |

| **Table S4.** Effects of substitution of macronutrients by saturated fatty acids (SFA), monounsaturated fatty acids (MUFA), polyunsaturated fatty acids (PUFA), protein and alcohol (per 5 energy percent) on Fatty Liver Index (FLI) (dependent variables)^*^ | | | |
| --- | --- | --- | --- |
| **Per 5% increased nutrient** | **ß-estimate** | **95% CI** | **p-value** |
| w/o SFA |  |  |  |
| Carbohydrates | 5.546 | (-2.361; 13.453) | 0.169 |
| MUFA | 16.654 | (0.156; 33.152) | ***0.048*** |
| PUFA | -17.263 | (-31.524; -3.002) | ***0.018*** |
| Protein | 31.921 | (20.566; 43.276) | ***<0.001*** |
| Alcohol | 6.255 | (-1.379; 13.889) | 0.108 |
| w/o MUFA |  |  |  |
| Carbohydrates | -11.395 | (-21.146; -1.645) | ***0.022*** |
| SFA | -17.919 | (-33.774; -2.065) | ***0.027*** |
| PUFA | -34.462 | (-57.886; -11.039) | ***0.004*** |
| Protein | 14.099 | (0.805; 27.394) | ***0.038*** |
| Alcohol | -10.270 | (-20.844; 0.305) | 0.057 |
| w/o PUFA |  |  |  |
| Carbohydrates | 18.942 | (2.553; 35.331) | ***0.024*** |
| SFA | 14.065 | (-0.241; 28.37) | 0.054 |
| PUFA | 28.981 | (4.53; 53.432) | ***0.020*** |
| Protein | 45.626 | (25.787; 65.465) | ***<0.001*** |
| Alcohol | 19.409 | (3.837; 34.982) | ***0.015*** |
| w/o Carbohydrates |  |  |  |
| SFA | -6.324 | (-14.391; 1.744) | 0.124 |
| MUFA | 11.482 | (1.129; 21.836) | ***0.030*** |
| PUFA | -23.332 | (-40.003; -6.662) | ***0.006*** |
| Protein | 26.121 | (18.825; 33.416) | ***<0.001*** |
| Alcohol | 0.775 | (-3.384; 4.934) | 0.715 |
| w/o Protein |  |  |  |
| Carbohydrates | -24.648 | (-31.495; -17.801) | ***<0.001*** |
| SFA | -31.074 | (-41.949; -20.199) | ***<0.001*** |
| MUFA | -14.014 | (-27.263; -0.765) | ***0.038*** |
| PUFA | -46.623 | (-65.562; -27.683) | ***<0.001*** |
| Alcohol | -23.113 | (-30.319; -15.906) | ***<0.001*** |
| w/o Alcohol |  |  |  |
| Carbohydrates | -0.954 | (-5.229; 3.321) | 0.661 |
| SFA | -7.201 | (-15.207; 0.805) | 0.078 |
| MUFA | 10.468 | (-1.072; 22.008) | 0.075 |
| PUFA | -24.107 | (-40.388; -7.826) | ***0.004*** |
| Protein | 25.161 | (17.269; 33.054) | ***<0.001*** |
| w/o Total fat |  |  |  |
| Carbohydrates | 0.128 | (-3.57; 3.826) | 0.946 |
| Protein | 25.148 | (16.014; 34.282) | ***<0.001*** |
| Alcohol | 1.955 | (-2.637; 6.548) | 0.403 |
| * Substitution models contained total energy intake, SFA, MUFA, PUFA, protein, and alcohol intake. Estimates are therefore interpreted as the association of FLI with a 5 E% increase in e.g. PUFA at the expense of carbohydrates while energy supply from other macronutrients remains unchanged; linear regression models adjusted for sex, age, physical activity, education years, smoking status and energy intake. CI, confidence interval | | | |
